# Analyses of key genes involved in Arctic adaptation in polar bears suggest selection on both standing variation and de novo mutations played an important role

**DOI:** 10.1186/s12864-020-06940-0

**Published:** 2020-08-06

**Authors:** Jose Alfredo Samaniego Castruita, Michael V. Westbury, Eline D. Lorenzen

**Affiliations:** GLOBE Institute, University of Copenhagen, Copenhagen, Denmark

**Keywords:** Polar bear, Adaptation, Arctic, Genomics, Selection

## Abstract

**Background:**

Polar bears are uniquely adapted to an Arctic existence. Since their relatively recent divergence from their closest living relative, brown bears, less than 500,000 years ago, the species has evolved an array of novel traits suited to its Arctic lifestyle. Previous studies sought to uncover the genomic underpinnings of these unique characteristics, and disclosed the genes showing the strongest signal of positive selection in the polar bear lineage. Here, we survey a comprehensive dataset of 109 polar bear and 33 brown bear genomes to investigate the genomic variants within these top genes present in each species. Specifically, we investigate whether fixed homozygous variants in polar bears derived from selection on standing variation in the ancestral gene pool or on de novo mutation in the polar bear lineage.

**Results:**

We find that a large number of sites fixed in polar bears are biallelic in brown bears, suggesting selection on standing variation. Moreover, we uncover sites in which polar bears are fixed for a derived allele while brown bears are fixed for the ancestral allele, which we suggest may be a signal of de novo mutation in the polar bear lineage.

**Conclusions:**

Our findings suggest that, among other mechanisms, natural selection acting on changes in genes derived from a combination of variation already in the ancestral gene pool, and from de novo missense mutations in the polar bear lineage, may have enabled the rapid adaptation of polar bears to their new Arctic environment.

## Background

The divergence of polar bears (*Ursus maritimus*) from brown bears (*Ursus arctos*) and their adaptation to a novel environment and lifestyle in the Arctic is a well-known example of rapid evolution [1, 2]. Despite having diverged relatively recently (ca. 479–343 thousand years ago (kya)) [1], polar bears evolved a novel and distinct ecology, behaviour, and morphology. This rapid evolution was proven even more substantial by stable isotope analysis of an ancient polar bear jawbone from Svalbard, indicating that the species had already adapted to a marine diet and life in the High Arctic by at least 110 kya [3]. Therefore, in perhaps as little as 20,000 generations, polar bears evolved a suite of unique adaptations that enable them to maintain homeostasis under low temperatures, occupy a hypercarnivorous niche, subsist on a diet of primarily seals and their blubber [4], process lipids as their predominant energy source [5, 6], and camouflage into their surroundings with pigment-free fur [7].

The rapid adaptation of such an iconic species has sparked a number of studies seeking to unravel the genomic underpinnings through the use of various datasets and analyses [1, 2, 8]. Both Miller et al. [2] and Liu et al. [1], investigated whole-genome datasets to uncover genomic regions showing signatures of selection. Together, these studies revealed different yet complementary sets of genes, pathways, and phenotypes that were likely shaped by natural selection during polar bear evolution. Genes showing the strongest signal of positive selection are involved in adipose tissue development, fatty acid metabolism, heart function, and fur pigmentation [1], indicating these are key processes in polar bear adaptation to the Arctic.

Using all currently available nuclear genomes from polar bears (*n* = 109) and brown bears (*n* = 33), we set out to determine the nature of the polar bear-specific amino acid changes in the top genes showing the strongest signal of positive selection in the polar bear lineage as reported by Liu et al., (Table 1 and supplementary Table S1) [1]. This increase in sample number and spatial coverage enabled us to more comprehensively investigate the origins of polar bear-specific substitutions. By comparing the target gene regions across the two species based on this comprehensive dataset, we (i) determine the number and location of missense substitutions specific to the polar bear lineage (ii) address whether the polar bear variants derive from standing variation or de novo mutations, (iii) evaluate whether the variants fixed in polar bears may lead to functional changes in the genes.

## Results

### Population structure and admixture

To gain a better understanding of whether selection acted upon standing variation in the polar/brown bear ancestral lineage or on de novo mutations in the polar bear lineage, we first needed to determine whether derived variants were already present in the ancestral gene pool. However, as admixture from polar bears into brown bears has been well documented [1, 2, 9–11], we first investigated admixture at our selected genes. If any introgression from polar bears persists in the brown bear gene pool, inferences of the origins of these variants (standing variation or de novo) may be biased. To do this, we ran independent PCAs on each of the twelve genes analysed including the 50 kb flanking regions up and downstream of the genes.

Clear genetic differentiation between polar bears and brown bears has been shown at a genome-wide scale [1, 2]. For eleven of the twelve genes, polar bears and brown bears form separate clusters, which we expect if there is no introgressive admixture/ILS at these loci (Supplementary Figs. 1–5 and 7–12). However, for EDH3, seven of the brown bears cluster within the diversity of polar bears, indicating some level of introgression or ILS around this gene (Supplementary Fig. 6). The seven brown bears were all from the Admiralty, Baranof, and Chichagof islands in Alaska, an area known to be inhabited by introgressed brown bears [9]. We therefore excluded EDH3 from further analysis.

### Non-synonymous differences between polar bears and brown bears

For this analysis, we only considered dinucleotide sites coding for non-synonymous amino acid changes. Firstly, we calculated the percentages of sites fixed within polar bears (Table 1), which ranged between 10 of 8424 coding sites (0.12%) in FCGBP to 18 of 3957 coding sites (0.45%) in LAMC3. We compared this to the number of sites also fixed in brown bears for an alternative allele. Seven out of the eleven genes contained sites showing this pattern (Table 1). More specific information regarding gene positions, amino acid changes, and allele counts can be found in supplementary Table S2.

**Table 1:** Number of sites in candidate genes showing the strongest signal of selection in polar bears

Gene	Associated phenotype	Coding length (bp)	Alternative allele in brown bear		Ancestral allele in brown bear	
			Standing variation	de novo	Standing variation	de novo
ABCC6*	Cardiovascular	4551	12	2	3	2
AIM1*	Pigmentation	5484	23	2	5	2
APOB*	Cardiovascular, metabolism, pigmentation	13,305	49	4	12	4
COL5A3*	Adipose tissue, metabolism	5256	16	4	2	1
CUL7	Adipose tissue, cardiovascular, metabolism	4308	13	0	2	0
FCGBP	NA	8424	10	0	0	0
LAMC3	NA	3957	18	0	2	0
LYST*	Metabolism, pigmentation	11,403	32	7	9	6
POLR1A*	Adipose tissue	5172	16	1	2	1
TTN*	Cardiovascular	102,861	117	20	16	12
XIRP1	Cardiovascular	5541	10	0	0	0

Putative Arctic-associated phenotypes are included for each gene; NA refers to genes in which no associated phenotype appears to be explicitly related to adaptations to the Arctic. Phenotype information was obtained from GeneCards (genecards.org). Sites fixed in polar bears are either biallelic (segregate with two alleles), fixed for an alternative allele, or fixed for the ancestral allele (also found in giant panda), in brown bears. A schematic showing the distribution of alleles (coloured dots) in the polar bear (light blue circle) and brown bear (light brown circle) gene pools has been included to ease interpretation. Blue dots represent the allele fixed in polar bears; brown dots are the alternative allele (with unknown ancestral state) in brown bears; white dots represent the ancestral allele (also found in giant panda). Asterisk indicates the seven genes that have sites putatively indicating de novo mutations: they are fixed in polar bears for the derived allele, and fixed in brown bears for the ancestral allele.

### Fixed derived missense mutations in polar bear

**Fig. 1:**
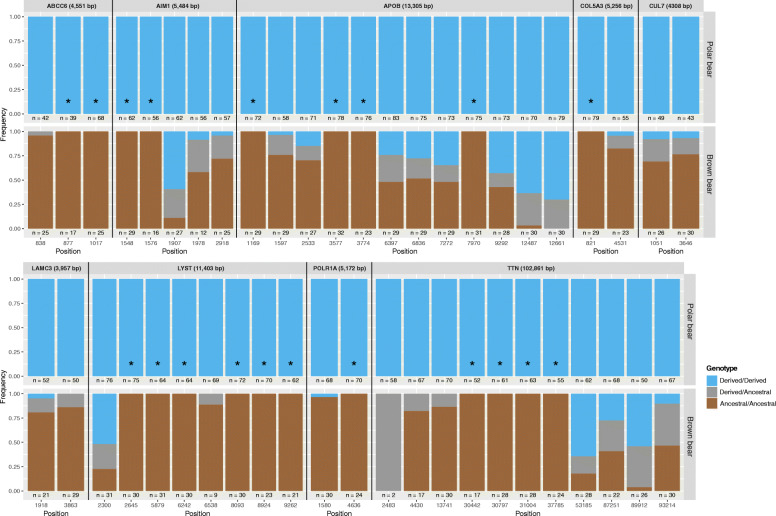
Relative proportions of genotype frequencies at biallelic sites across nine genes with fixed variants in the polar bear lineage. Only sites which are fixed in polar bears for the derived allele and are biallelic or fixed in brown bears for the ancestral allele are shown. Sites fixed for the derived allele in the polar bear lineage and fixed for the ancestral allele in the brown bear lineage (putative de novo mutations) are indicated with an asterisk. Gene length and the position of each site along the gene is indicated, as are the number of individuals (n = X) with more than 4x coverage at said site

To understand the origins of the variants fixed in the polar bear lineage relative to brown bears (i.e. whether they are derived or ancestral), we compared the polar bear and brown bear alleles to the giant panda (*Ailuropoda melanoleuca*) reference sequence, assuming the ancestral variant is retained in the panda. This was to reduce any noise caused by changes occurring in the brown bear lineage after its divergence from polar bears. Therefore, we only included sites where polar bears acquired a derived allele, while brown bears retained the ancestral state. Seven of the eleven genes of interest had at least one site showing this pattern (Table 1, Fig. 1). The number of such sites varied from zero (CUL7, FCGBP, LAMC3, and XIRP1) to twelve (TTN). More specific information regarding the gene positions and allele counts can be found in supplementary Table S3.

### Influence on protein structure

To evaluate whether the fixed derived amino acid changes in polar bears (also fixed for the ancestral allele in brown bears) had any functional influence on the protein function, we implemented three independent analyses to predict the functional effects of the amino acid change in humans. Amino acid changes that appeared to have a functional influence according to at least one of the three tests were found in four genes, with varying numbers of sites displaying this in each gene, TTN (9 sites), LYST (3 sites), AIM1 (2 sites), and COL5A3 (1 site) (Supplementary Table S3). However, none of these sites showed consistent deleterious effects across all three analyses. All other amino acid changes were deemed either benign, neutral or tolerated. It should be noted that these amino acid changes may have a different influence on polar bear proteins compared to humans, and this should be taken into account in the interpretation of these results.

## Discussion

To increase our understanding of how polar bears adapted to the Arctic, we investigated the origins of the genomic variants in eleven candidate genes previously found to show the strongest signals of positive selection in the polar bear lineage [1], nine of which have functions that could be linked to Arctic-specific adaptations, including adipose tissue development, fatty acid metabolism, heart function, and fur pigmentation (Table 1). We analysed a comprehensive panel of polar bear and brown bear nuclear genomes, and identified biallelic sites in which alternative alleles result in an amino acid change within these genes.

We find the majority of sites fixed in polar bears are biallelic in brown bears (Table 1, Fig. 1). This result may reflect that natural selection more readily acted upon standing variation already in the ancestral polar/brown bear gene pool, allowing for more rapid adaptation compared to selection on de novo mutations. However, we also identify a number of sites in seven of the eleven genes analysed, in which polar bears are fixed for the derived allele and brown bears are fixed for the alternative, ancestral allele, relative to the outgroup giant panda. Although the absolute number of sites showing this pattern is relatively low (ranging from 1 to 12 sites in each gene), these findings suggest selection may also have acted on de novo mutations in the polar bear lineage.

However, our results may also reflect the relatively low genetic diversity in polar bears compared to brown bears (Supplementary Figs. 1–12), which is due to the long-term, low effective population size of polar bears [1, 2]. Such low diversity would have exacerbated the impact of genetic drift on standing variation, potentially leading to the fixation of variable sites in the polar bear lineage. In contrast, it is unlikely that de novo mutations reached fixation by genetic drift alone, even in the fairly homogenous polar bear gene pool. The initial low frequency of a de novo mutation would more likely lead to the mutation being purged from the gene pool rather than being fixed via genetic drift. Therefore, it is more probable that the de novo polar bear-specific substitutions reached fixation due to strong selective pressures, as opposed to simply reflecting the relatively limited genetic diversity of polar bears relative to brown bears.

Moreover, at both variant types (standing variation and de novo), we find evidence for protein structure-altering substitutions in the polar bear lineage (Supplementary Table S3), potentially leading to phenotypic change in the species. This adds weight to our hypothesis that selection acted on both standing variation and on de novo mutations. However, we cannot exclude that the fixation of some of these phenotypically relevant mutations may be due to genetic hitchhiking from one causative mutation in the gene. Regardless, the fact that putatively phenotypic changes occur at both variant types suggests natural selection acted upon both standing variation and de novo mutations.

The genes with the highest proportion of sites indicating de novo mutations were APOB and ABCC6 (with four and two such sites, respectively), which are associated with the cardiovascular system, and LYST and AIM1 (with seven and two such sites, respectively), which are associated with pigmentation (Table 1).

The APOB gene codes for apolipoprotein B (apoB), the primary lipid-binding protein of chylomicrons and low-density lipoproteins (LDL), which enables the mobility of fat molecules around the body [12, 13]. The ABCC6 gene encodes for a protein belonging to the superfamily of ATP-binding cassette (ABC) transporters and is involved in transporting various molecules across extra- and intra-cellular membranes. Diseases associated with ABCC6 include Pseudoxanthoma elasticum (PXE), a neurocutaneous disorder that affects the elastic tissue of the cardiovascular system, causes arterial calcification, and increases the risk of coronary artery disease [14–16]. Selection on the APOB and ABCC6 genes may have played a role in the novel adaptation of polar bears to a lipid-rich diet, and increased the efficacy of cholesterol clearance from the blood [1].

The LYST gene codes for the lysosomal trafficking regulator Lyst. Mutations in the LYST gene have been reported to cause hypopigmentation, a melanosome defect characterized by light coat color [17, 18]. AIM1 is also associated with colouration, as variable expression of AIM1 has been associated with tumor suppression in human melanoma, influencing melanin pigment production [19]. Selection on LYST and AIM1 may have led to the lack of fur pigmentation in polar bears, resulting in the characteristic white phenotype of the species that may confer a selective advantage in the Arctic. Detrimental amino acid changes may have significantly hindered the function of these genes, resulting in the lack of pigmentation for natural selection to act upon.

## Conclusion

Although genes involved in adaptation of polar bears to their Arctic lifestyle have previously been uncovered [1, 2], a comprehensive assessment of whether selection on these genes acted upon standing variation or de novo mutations has been lacking. In the present study, through the analysis of a comprehensive data set of polar bear and brown bear genomes, we were able to address this question and provide new insights into the origins of variants found in genes under selection (did they derive from standing variation in the ancestral gene pool or de novo mutation?) that putatively enabled the rapid adaptation in polar bears to the Arctic.

## Methods

For this study, we analysed publicly available whole-genome data from 109 polar bears and 33 brown bears (Supplementary Table S4). This included the data set from Liu et al. [1] of 89 genomes and an additional 30 polar bear and 23 brown bear genomes published elsewhere [2, 9, 11, 20, 21]. We downloaded SRA files from NCBI (Bioproject IDs: PRJNA169236, PRJNA196978, PRJNA210951, PRJNA271471, PRJNA395974, and PRJEB27491). Polar bear individuals originated from West Greenland, East Greenland, Canada, Siberia, USA (Alaska), and Svalbard (genome-wide coverage of 1.7x - 114.1x). Brown bear individuals originated from the USA (Montana, Alaska), Sweden, Finland, Italy, Greece, Slovakia, Spain, Slovenia, Georgia, and Russia (genome-wide coverage of 2.7x - 58.2x). The recently published brown bear reference genome individual was excluded from the analysis due to unknown provenance [22].

### Raw data processing

We processed all raw sequencing reads with the PALEOMIX [23] pipeline. Internally, adapter sequences, stretches of Ns, and low-quality bases were trimmed and filtered with AdapterRemovalv2 [24] using default parameters. BWA-backtrack v0.7.15 [25] was used to map the cleaned reads to the pseudo-chromosomal polar bear genome (Genbank accession: GCA_000687225.1) from Liu et al. [1], with default parameters. Reads with mapping quality of less than 30 were filtered using SAMtools v1.6 [26]. Duplicates were removed with picard v2.6.0 [27]. Possible paralogs were filtered using SAMtools. Finally, local realignment around indels was performed using GATK (v 3.3) [28].

### Population structure and admixture

To investigate whether admixture or incomplete lineage sorting (ILS) may be present between polar bears and brown bears at the genes of interest, we performed independent principal component analyses (PCAs) for each gene including the 50 kb regions up and downstream of the gene. For this, we included all polar and brown bear individuals. We used a genotype likelihood approach to construct the PCAs: input genotype likelihood files were constructed using ANGSD v0.929 [29], with the SAMtools genotype likelihood algorithm (−GL 1), and specifying the following parameters: remove reads that have multiple mapping best hits (−unique_only), remove reads with a flag above 255/secondary hits (−remove_bads), include only read pairs with both mates mapping correctly (−only_proper_pairs), adjust mapQ for reads with excessive mismatches (−C 50), adjust quality scores around indels (−baq 1), a minimum mapping quality of 20 (−minMapQ 20), a minimum base quality of 20 (−minQ 20), discard sites where there is no data on at least 95% of the individuals (−minInd), skip tri-allelic sites (−skipTriallelic), and remove SNP sites with a *p*-value larger than 1e^− 6^ (−SNP_pval 1e-6). The ANGSD output beagle file was run through PCAngsd v0.95 [30].

### Gene investigation

We analysed twelve of the genes previously found to show the strongest signal of positive selection in the polar bear [1]. These included ABCC6, AIM1, APOB, COL5A3, CUL7, EHD3, FCGBP, LAMC3, LYST, POLR1A, TTN, and XIRP1. The phenotypes putatively associated with these genes can be found in Table 1 and supplementary Table S1.

Genotypes were called using ANGSD, specifying the same parameters as the PCA analyses with the additional parameters: write major and minor alleles and the genotype directly (−doGeno 5), estimate the posterior genotype probability based on the allele frequency as a prior (−doPost 1), use the reference allele as the major allele (−doMajorMinor 4), output as beagle likelihood file (−doGlf 2), and calculate allele frequencies assuming a fixed major allele and an unknown minor allele (−doMaf 2). In order to decrease biases that could arise when calling heterozygous alleles from the low coverage genomes, we only called genotypes from individuals that had at least 4x coverage at the site of interest (−geno_minDepth 4). For the investigation into which alleles represent the ancestral variant, we downloaded each of the relevant giant panda gene transcript sequences from Genbank (Supplementary Table S5). Although assembled genome data is available from Ursidae species more closely related to the polar bear and brown bear (e.g. black bear [31]), we specifically avoided the use of other Ursine bears to determine the ancestral state, as introgressive gene flow has been identified across nearly all species of Ursine bears [32] which may lead to the misidentification of the ancestral variant. We additionally included genomic data from a ~ 110 k year old polar bear sample from Svalbard [3] (NCBI Bioproject ID: PRJNA169236) in an attempt to understand when the variants may have arisen. However, due to the very low coverage (~ 0.34x) manner of the Poolepynten bear, we did not recover any sites with enough data to incorporate into our analyses.

We performed predictions of the effects of amino acid changes found in putative de novo mutations on the polar bear lineage on the function of homologous human proteins using Polyphen-2 [33], SIFT [34], and PROVEAN [35]. For this analysis we downloaded the relevant human gene transcript sequences from Genbank (Supplementary Table S5). This was done by aligning the human and polar bear protein genes and selecting only the positions that were fixed for the derived allele in polar bears and fixed for the ancestral allele (shared allele with the giant panda) in brown bears. Positions and amino acid changes were submitted to Polyphen-2 batch web service using HumDiv and HumVar model classifiers as well as to PROVEAN human protein batch web service for the PROVEAN and SIFT analyses.

## Supplementary information

**Additional file 1: Supplementary Table S1.** Phenotypes associated with the genes of interest. Phenotype information taken from GeneCards (genecards.org). * UniProtKB/Swiss-Prot summary as the associated phenotypes are not available on Genecards. **Supplementary Table S5:** Genbank accession codes for the polar bear, giant panda (annotation version 102*), and human transcript sequences used in the study. *Available from: https://ftp.ncbi.nlm.nih.gov/genomes/all/annotation_releases/9646/102/. **Supplementary Fig. 1:** Principal component analysis of ABCC6 and the 50 kb flanking regions using all individuals included in this study. **Supplementary Fig. 2:** Principal component analysis of AIM1 and the 50 kb flanking regions using all individuals included in this study. **Supplementary Fig. 3:** Principal component analysis of APOB and the 50 kb flanking regions using all individuals included in this study. **Supplementary Fig. 4:** Principal component analysis of COL5A and the 50 kb flanking regions using all individuals included in this study. **Supplementary Fig. 5:** Principal component analysis of CUL7 and the 50 kb flanking regions using all individuals included in this study. **Supplementary Fig. 6:** Principal component analysis of EHD3 and the 50 kb flanking regions using all individuals included in this study. **Supplementary Fig. 7:** Principal component analysis of FCGBP and the 50 kb flanking regions using all individuals included in this study. **Supplementary Fig. 8:** Principal component analysis of LAMC3 and the 50 kb flanking regions using all individuals included in this study. **Supplementary Fig. 9:** Principal component analysis of LYST and the 50 kb flanking regions using all individuals included in this study. **Supplementary Fig. 10:** Principal component analysis of POLR1A and the 50 kb flanking regions using all individuals included in this study. **Supplementary Fig. 11:** Principal component analysis of TTN and the 50 kb flanking regions using all individuals included in this study. **Supplementary Fig. 12:** Principal component analysis of XIRP1 and the 50 kb flanking regions using all individuals included in this study.

**Additional file 2: Supplementary Table S2.** Table including all relevant information at the genes of interest, including nucleotide information, gene location, amino acid information, and allele distributions of the non-synonymous differences between polar bears and brown bears.

**Additional file 3: Supplementary Table S3.** Table including all relevant information at the genes of interest, including nucleotide information, gene location, amino acid information, allele distributions, and predictions of amino acid changes in the sites in which the alternative allele is also found in the giant panda. Red coloured cells indicate sites in which amino acid changes may have a functional influence according to at least one of the three tests: Polyphen-2, SIFT, or PROVEAN. D/D represents homozygous derived, D/A represents heterozygous derived and ancestral, A/A represents homozygous ancestral.

**Additional file 4: Supplementary Table S4.** Polar bear and brown bear sample locality and mapping information.

## Data Availability

All polar and brown bear short read data (including the ancient Poolepynten bear) can be found under the following NCBI Bioproject IDs: PRJNA169236, PRJNA196978, PRJNA210951, PRJNA271471, PRJNA395974, and PRJEB27491. The polar bear genome used as the mapping reference can be found under the Genbank assembly accession number: GCA_000687225.1 found under the following link: https://www.ncbi.nlm.nih.gov/assembly/GCF_000687225.1/. The pseudo-chromosome version of the above polar bear genome produced by Liu et al. 2013, can be found on the University of Copenhagen’s Electronic Research Data Archive (ERDA) under the following link: https://sid.erda.dk/share_redirect/DAyPNtKCOV. Genbank accession codes for all of the polar bear, giant panda, and human transcripts used in this study can be found in supplementary Table S5.

## Abbreviations

PB: Polar bear
BB: Brown bear
PCA: Principal component analysis
ILS: Incomplete lineage sorting
kya: Thousand years ago
bp: Base pairs
apoB: Apolipoprotein B
LDL: Low-density lipoproteins
ABC: ATP-binding cassette
PXE: Pseudoxanthoma elasticum

## References

[1] Liu S, Lorenzen ED, Fumagalli M, Li B, Harris K, Xiong Z (2014). Population genomics reveal recent speciation and rapid evolutionary adaptation in polar bears. Cell..

[2] Miller W, Schuster SC, Welch AJ, Ratan A, Bedoya-Reina OC, Zhao F (2012). Polar and brown bear genomes reveal ancient admixture and demographic footprints of past climate change. Proc Natl Acad Sci U S A.

[3] Lindqvist C, Schuster SC, Sun Y, Talbot SL, Qi J, Ratan A (2010). Complete mitochondrial genome of a Pleistocene jawbone unveils the origin of polar bear. Proc Natl Acad Sci U S A.

[4] Thiemann GW, Iverson SJ, Stirling I (2008). Polar bear diets and arctic marine food webs: insights from fatty acid analysis. Ecol Monogr.

[5] Derocher AE, Wiig Ø, Andersen M (2002). Diet composition of polar bears in Svalbard and the western Barents Sea. Polar Biol.

[6] Best RC (1985). Digestibility of ringed seals by the polar bear. Can J Zool.

[7] Harington CR (2008). The evolution of Arctic marine mammals. Ecol Appl.

[8] Rinker DC, Specian NK, Zhao S, Gibbons JG (2019). Polar bear evolution is marked by rapid changes in gene copy number in response to dietary shift. Proc Natl Acad Sci U S A.

[9] Cahill JA, Green RE, Fulton TL, Stiller M, Jay F, Ovsyanikov N (2013). Genomic evidence for island population conversion resolves conflicting theories of polar bear evolution. PLoS Genet.

[10] Cahill JA, Heintzman PD, Harris K, Teasdale MD, Kapp J, Soares AER (2018). Genomic evidence of widespread admixture from polar bears into Brown bears during the last ice age. Mol Biol Evol.

[11] Cahill JA, Stirling I, Kistler L, Salamzade R, Ersmark E, Fulton TL (2015). Genomic evidence of geographically widespread effect of gene flow from polar bears into brown bears. Mol Ecol.

[12] Benn M (2009). Apolipoprotein B levels, APOB alleles, and risk of ischemic cardiovascular disease in the general population, a review. Atherosclerosis..

[13] Whitfield AJ, Barrett PHR, van Bockxmeer FM, Burnett JR (2004). Lipid disorders and mutations in the APOB gene. Clin Chem.

[14] Trip MD, Smulders YM, Wegman JJ, Hu X, Boer JMA, ten Brink JB (2002). Frequent mutation in the ABCC6 gene (R1141X) is associated with a strong increase in the prevalence of coronary artery disease. Circulation..

[15] Nitschke Y, Baujat G, Botschen U, Wittkampf T, du Moulin M, Stella J (2012). Generalized arterial calcification of infancy and pseudoxanthoma elasticum can be caused by mutations in either ENPP1 or ABCC6. Am J Hum Genet.

[16] Miksch S, Lumsden A, Guenther UP, Foernzler D, Christen-Zäch S, Daugherty C (2005). Molecular genetics of pseudoxanthoma elasticum: type and frequency of mutations in ABCC6. Hum Mutat.

[17] Runkel F, Büssow H, Seburn KL, Cox GA, Ward DM, Kaplan J (2006). Grey, a novel mutation in the murine Lyst gene, causes the beige phenotype by skipping of exon 25. Mamm Genome.

[18] Gutiérrez-Gil B, Wiener P, Williams JL (2007). Genetic effects on coat colour in cattle: dilution of eumelanin and phaeomelanin pigments in an F2-backcross Charolais× Holstein population. BMC Genet.

[19] Trent JM, Stanbridge EJ, McBride HL, Meese EU, Casey G, Araujo DE (1990). Tumorigenicity in human melanoma cell lines controlled by introduction of human chromosome 6. Science..

[20] Benazzo A, Trucchi E, Cahill JA, Maisano Delser P, Mona S, Fumagalli M (2017). Survival and divergence in a small group: the extraordinary genomic history of the endangered Apennine brown bear stragglers. Proc Natl Acad Sci U S A.

[21] Barlow A, Cahill JA, Hartmann S, Theunert C, Xenikoudakis G, Fortes GG (2018). Partial genomic survival of cave bears in living brown bears. Nat Ecol Evol.

[22] Taylor GA, Kirk H, Coombe L, Jackman SD, Chu J, Tse K, et al. The Genome of the North American Brown Bear or Grizzly: *Ursus arctos ssp. horribilis* . Genes [Internet]. 2018 Nov 30;9(12). Available from: 10.3390/genes9120598. 10.3390/genes9120598 PMC6315469 30513700

[23] Schubert M, Ermini L, Der Sarkissian C, Jónsson H, Ginolhac A, Schaefer R (2014). Characterization of ancient and modern genomes by SNP detection and phylogenomic and metagenomic analysis using PALEOMIX. Nat Protoc.

[24] Schubert M, Lindgreen S, Orlando L (2016). AdapterRemoval v2: rapid adapter trimming, identification, and read merging. BMC Res Notes.

[25] Li H, Durbin R (2009). Fast and accurate short read alignment with burrows–wheeler transform. Bioinformatics..

[26] Li H, Handsaker B, Wysoker A, Fennell T, Ruan J, Homer N (2009). The sequence alignment/map format and SAMtools. Bioinformatics..

[27] Broad institute. Picard Toolkit [Internet]. 2019. Available from: http://broadinstitute.github.io/picard.

[28] McKenna A, Hanna M, Banks E, Sivachenko A, Cibulskis K, Kernytsky A (2010). The genome analysis toolkit: a MapReduce framework for analyzing next-generation DNA sequencing data. Genome Res.

[29] Korneliussen TS, Albrechtsen A, Nielsen R (2014). ANGSD: analysis of next generation sequencing data. BMC Bioinformatics.

[30] Meisner J, Albrechtsen A (2018). Inferring population structure and admixture proportions in low-depth NGS data. Genetics..

[31] Srivastava A, Kumar Sarsani V, Fiddes I, Sheehan SM, Seger RL, Barter ME (2019). Genome assembly and gene expression in the American black bear provides new insights into the renal response to hibernation. DNA Res.

[32] Kumar V, Lammers F, Bidon T, Pfenninger M, Kolter L, Nilsson MA (2017). The evolutionary history of bears is characterized by gene flow across species. Sci Rep.

[33] Adzhubei IA, Schmidt S, Peshkin L, Ramensky VE, Gerasimova A, Bork P (2010). A method and server for predicting damaging missense mutations. Nat Methods.

[34] Sim N-L, Kumar P, Hu J, Henikoff S, Schneider G, Ng PC. SIFT web server: predicting effects of amino acid substitutions on proteins. Nucleic Acids Res. 2012 Jul;40(Web Server issue):W452–7. 10.1093/nar/gks539 PMC3394338 22689647

[35] Choi Y, Chan AP (2015). PROVEAN web server: a tool to predict the functional effect of amino acid substitutions and indels. Bioinformatics..

