# Supplementary material for: Analyses of key genes involved in Arctic adaptation in polar bears suggest selection on both standing variation and de novo mutations played an important role
Source: BMC Genomics. 2020 Aug 6;21:543. doi: 10.1186/s12864-020-06940-0 (PMC7430819; doi:10.1186/s12864-020-06940-0)
Supplement: Supplementary file 1 — Additional file 1: Supplementary Table S1. Phenotypes associated with the genes of interest. Phenotype information taken from GeneCards (genecards.org). * UniProtKB/Swiss-Prot summary as the associated phenotypes are not available on Genecards. Supplementary Table S5: Genbank accession codes for the polar bear, giant panda (annotation version 102*), and human transcript sequences used in the study. *Available from: https://ftp.ncbi.nlm.nih.gov/genomes/all/annotation_releases/9646/102/. Supplementary Fig. 1: Principal component analysis of ABCC6 and the 50 kb flanking regions using all individuals included in this study. Supplementary Fig. 2: Principal component analysis of AIM1 and the 50 kb flanking regions using all individuals included in this study. Supplementary Fig. 3: Principal component analysis of APOB and the 50 kb flanking regions using all individuals included in this study. Supplementary Fig. 4: Principal component analysis of COL5A and the 50 kb flanking regions using all individuals included in this study. Supplementary Fig. 5: Principal component analysis of CUL7 and the 50 kb flanking regions using all individuals included in this study. Supplementary Fig. 6: Principal component analysis of EHD3 and the 50 kb flanking regions using all individuals included in this study. Supplementary Fig. 7: Principal component analysis of FCGBP and the 50 kb flanking regions using all individuals included in this study. Supplementary Fig. 8: Principal component analysis of LAMC3 and the 50 kb flanking regions using all individuals included in this study. Supplementary Fig. 9: Principal component analysis of LYST and the 50 kb flanking regions using all individuals included in this study. Supplementary Fig. 10: Principal component analysis of POLR1A and the 50 kb flanking regions using all individuals included in this study. Supplementary Fig. 11: Principal component analysis of TTN and the 50 kb flanking regions using all individuals included in this study. Supple [file 12864_2020_6940_MOESM1_ESM.docx]

**Supplementary tables:**

**Supplementary table S1:** Phenotypes associated with the genes of interest. Phenotype information taken from GeneCards (genecards.org). * UniProtKB/Swiss-Prot summary as the associated phenotypes are not available on Genecards

| **Gene name** | **Phenotypes with a functional association on GeneCards** | **Putative polar bear/Arctic association** |
| --- | --- | --- |
| ABCC6 | Cardiovascular system, integument, homeostasis/metabolism, craniofacial, renal/urinary system, vision/eye, growth/size/body region | Cardiovascular system |
| AIM1 | Not available (May function as suppressor of malignant melanoma. It may exert its effects through interactions with the cytoskeleton*) | Pigmentation |
| APOB | Homeostasis/metabolism, mortality/aging, nervous system, growth/size/body region, liver/biliary system, cardiovascular system, embryo, vision/eye, muscle, reproductive system, pigmentation, cellular, immune system | Metabolism, cardiovascular system, pigmentation |
| COL5A3 | Integument, adipose tissue, behavior/neurological, muscle, endocrine/exocrine gland, growth/size/body region, homeostasis/metabolism, cellular | Adipose tissue, metabolism |
| CUL7 | Growth/size/body region, mortality/aging, cardiovascular system, cellular, homeostasis/metabolism, embryo, no phenotypic, adipose tissue, respiratory system, skeleton | Cardiovascular system, metabolism, adipose tissue |
| EHD3 | Homeostasis/metabolism, cellular, cardiovascular system, renal/urinary system, mortality/aging, growth/size/body region | Cardiovascular system |
| FCGBP | Endocrine/exocrine, reproductive system, vision/eye | - |
| LAMC3 | Nervous system, eye/vision, immune system, hematopoietic system | - |
| LYST | Pigmentation, integument, immune system, vision/eye, hematopoietic system, hearing/vestibular/ear, growth/size/body region, limbs/digits/tail, craniofacial, behavior/neurological, nervous system, homeostasis/metabolism, cellular, neoplasm, mortality/aging, endocrine/exocrine gland, renal/urinary system, respiratory system | Pigmentation, metabolism |
| POLR1A | Growth/size/body region, adipose tissue, cellular, mortality/aging, embryo | Adipose tissue |
| TTN | Muscle, cardiovascular system, mortality/aging, homeostasis/metabolism, growth/size/body region, behavior/neurological, embryo, skeleton, cellular, immune system, craniofacial, vision/eye phenotype | Cardiovascular system, |
| XIRP1 | Cardiovascular system, muscle, growth/size/body region | Cardiovascular system |

**Supplementary table S5:** Genbank accession codes for the polar bear, giant panda (annotation version 102*) , and human transcript sequences used in the study.

*Available from: <https://ftp.ncbi.nlm.nih.gov/genomes/all/annotation_releases/9646/102/>

| **Gene** | **Polar Bear** | **Giant Panda** | **Human** |
| --- | --- | --- | --- |
| ABCC6 | XM_008696932.1 | XP_002927050.1 | NP_001162.4 |
| AIM1 | XM_008705044.1 | XP_011230960.2 | NP_001615.2 |
| APOB | XM_008700590.1 | XP_002930154.1 | NP_000375.2 |
| COL5A3 | XM_008708137.1 | XP_011225983.1 | NP_056534.2 |
| CUL7 | XM_008691977.1 | XP_002914522.1 | NP_001161842.1 |
| EHD3 | XM_008700558.1 | XP_011232801.1 | NP_055415.1 |
| FCGBP | XM_008688765.1 | XP_019659876.1 | NP_003881.2 |
| LAMC3 | XM_008698869.1 | XP_019650859.1 | NP_006050.3 |
| LYST | XM_008689569.1 | XP_011234392.1 | NP_000072.2 |
| POLR1A | XM_008700085.1 | XP_002928679.1 | NP_056240.2 |
| TTN | XM_008687526.1 | XP_019654795.1 | NP_001254479.2 |
| XIRP1 | XM_008710061.1 | XP_011215162.1 | NP_919269.2 |

**Supplementary Figures:**

**
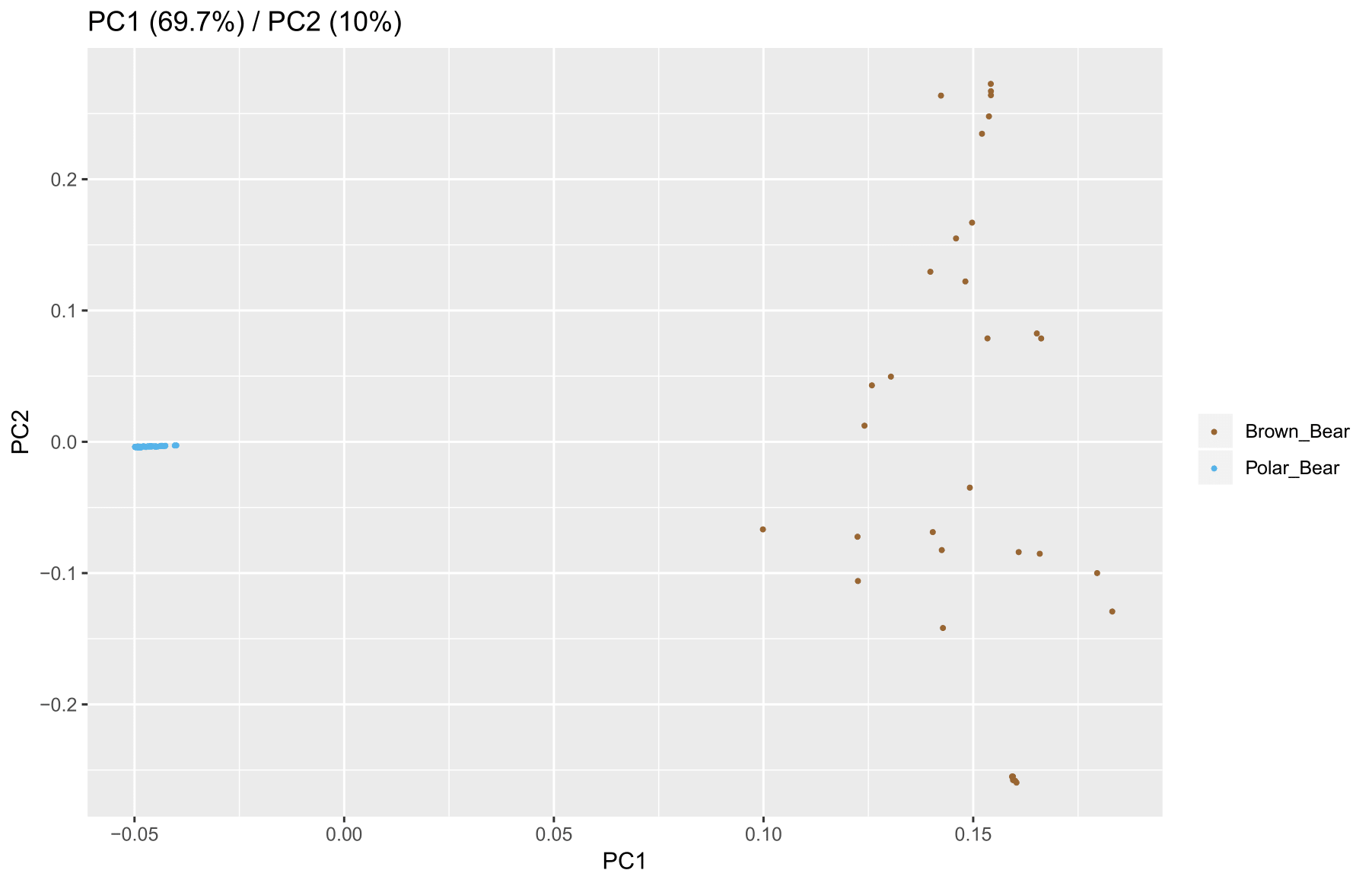
**

**Supplementary figure 1:** Principal component analysis of ABCC6 and the 50kb flanking regions using all individuals included in this study.

**
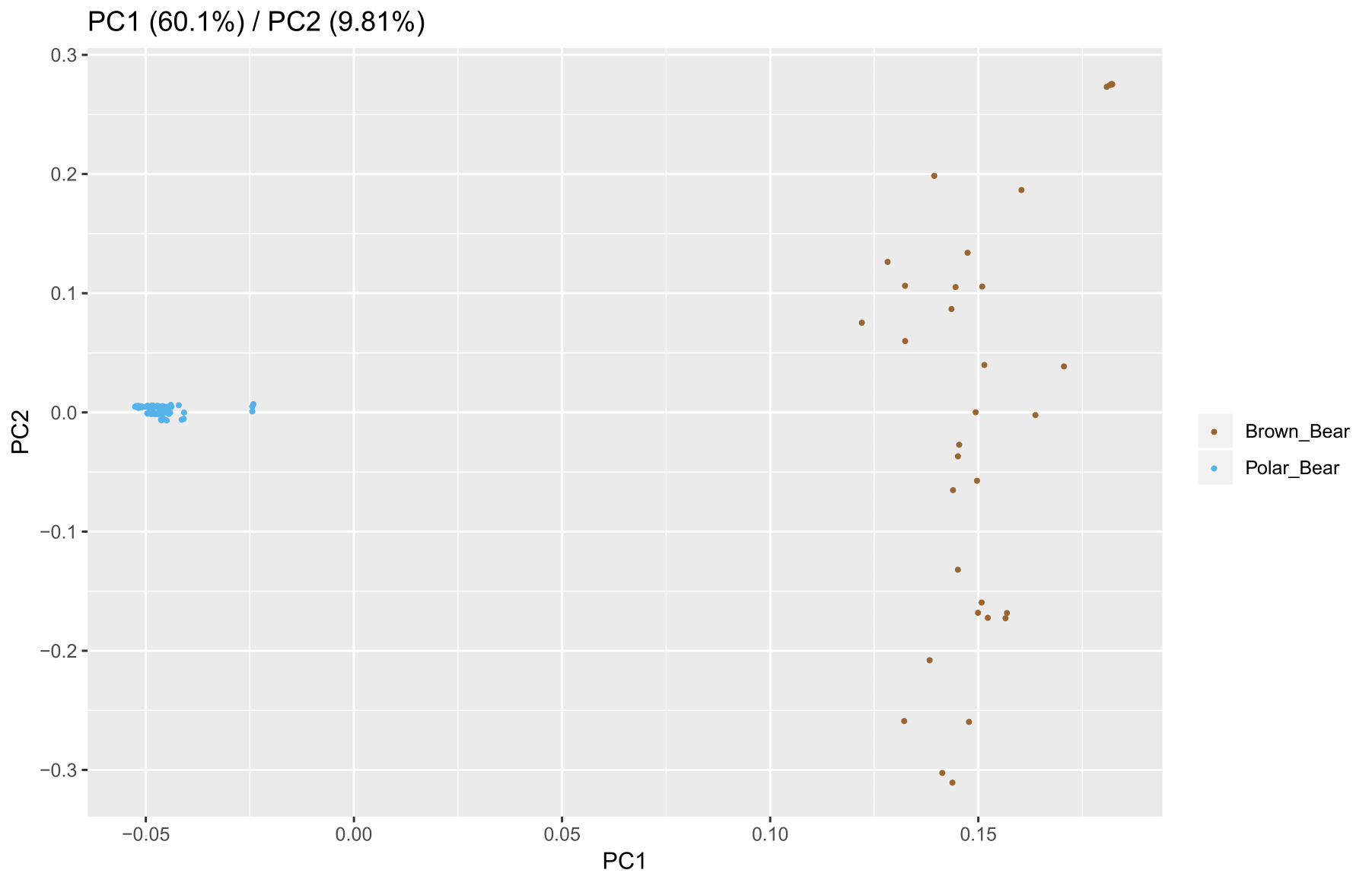
**

**Supplementary figure 2:** Principal component analysis of AIM1 and the 50kb flanking regions using all individuals included in this study.

**
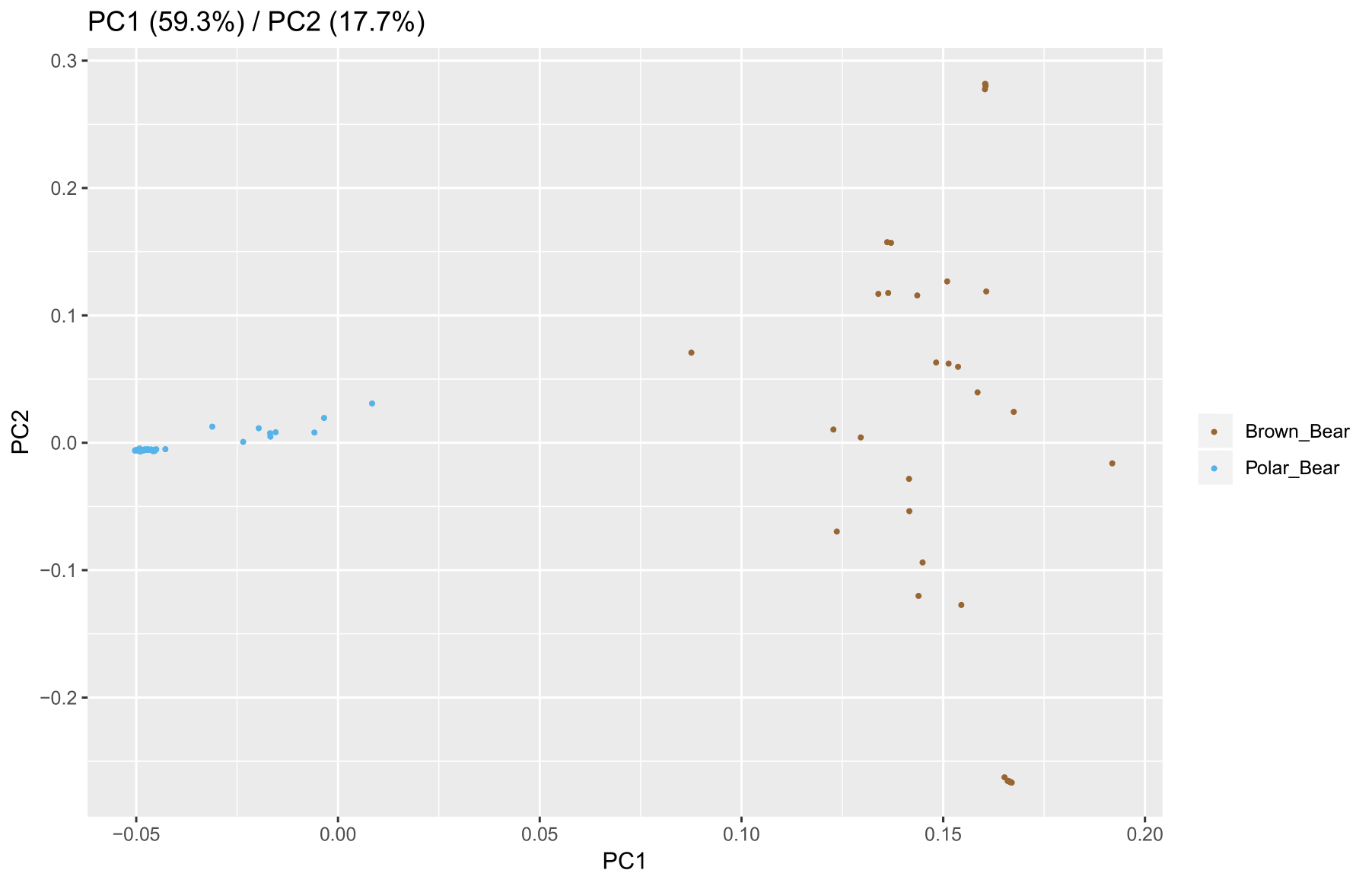
**

**Supplementary figure 3:** Principal component analysis of APOB and the 50kb flanking regions using all individuals included in this study.

**
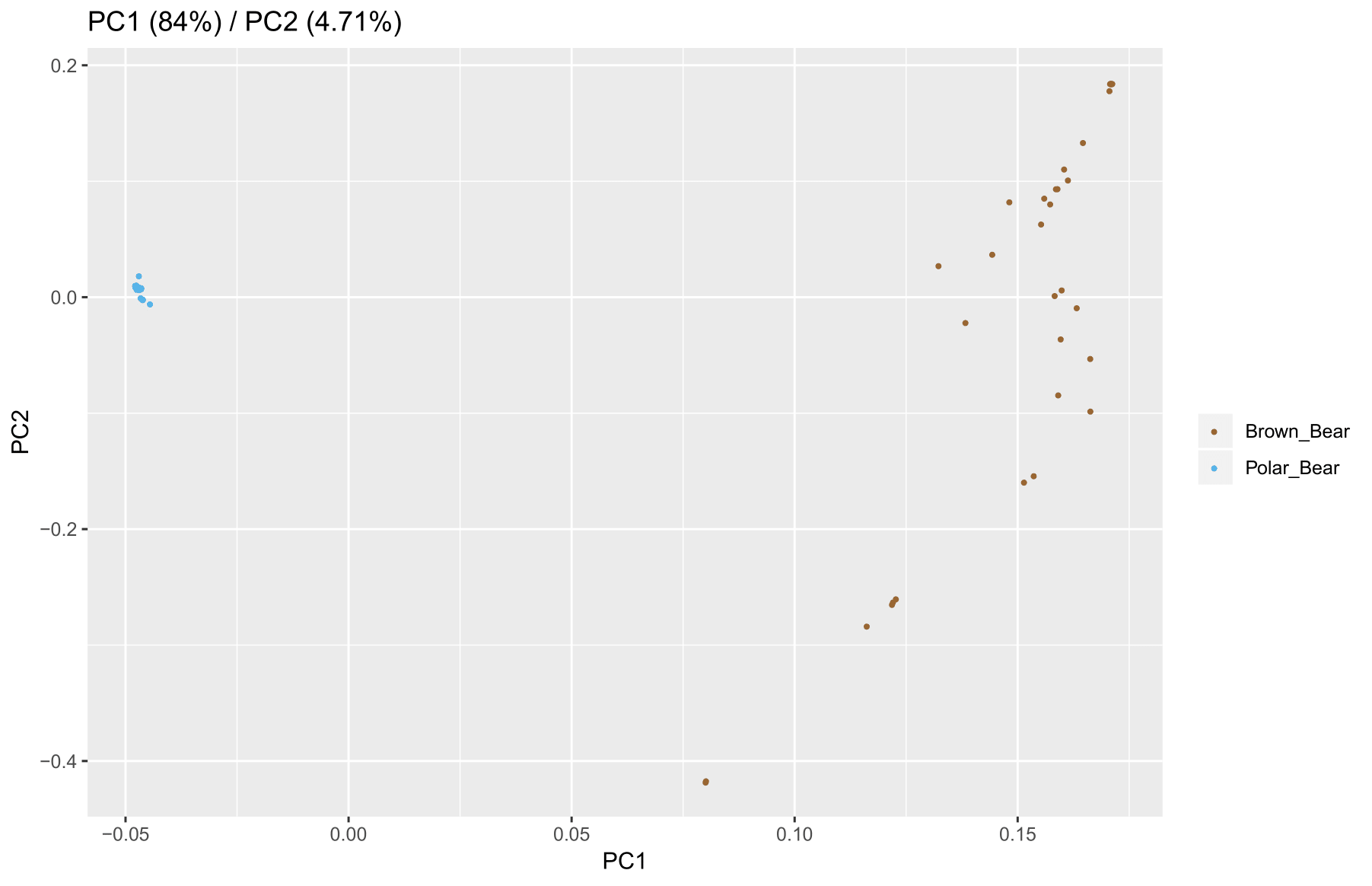
**

**Supplementary figure 4:** Principal component analysis of COL5A and the 50kb flanking regions using all individuals included in this study.

**
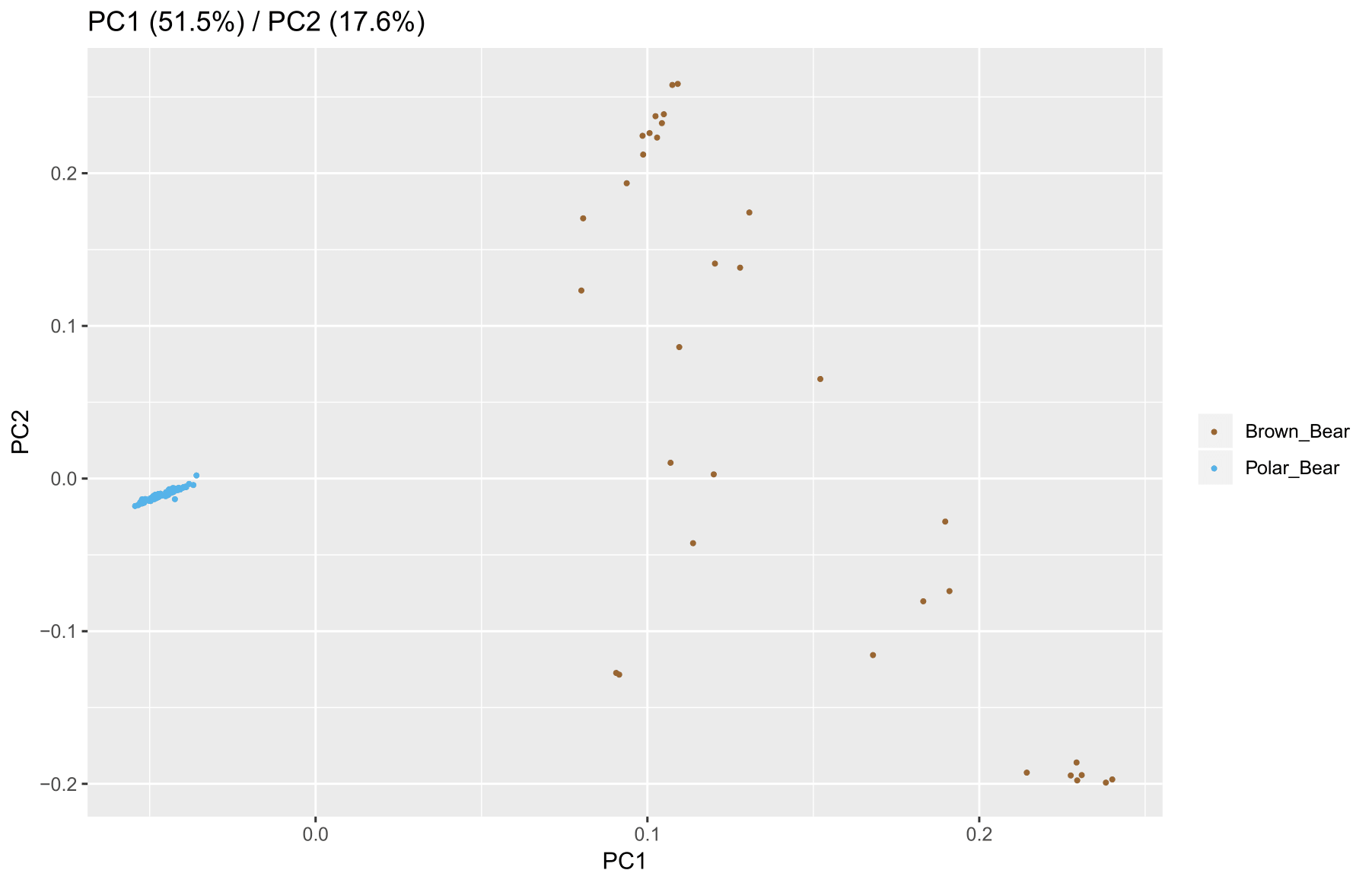
**

**Supplementary figure 5:** Principal component analysis of CUL7 and the 50kb flanking regions using all individuals included in this study.

**
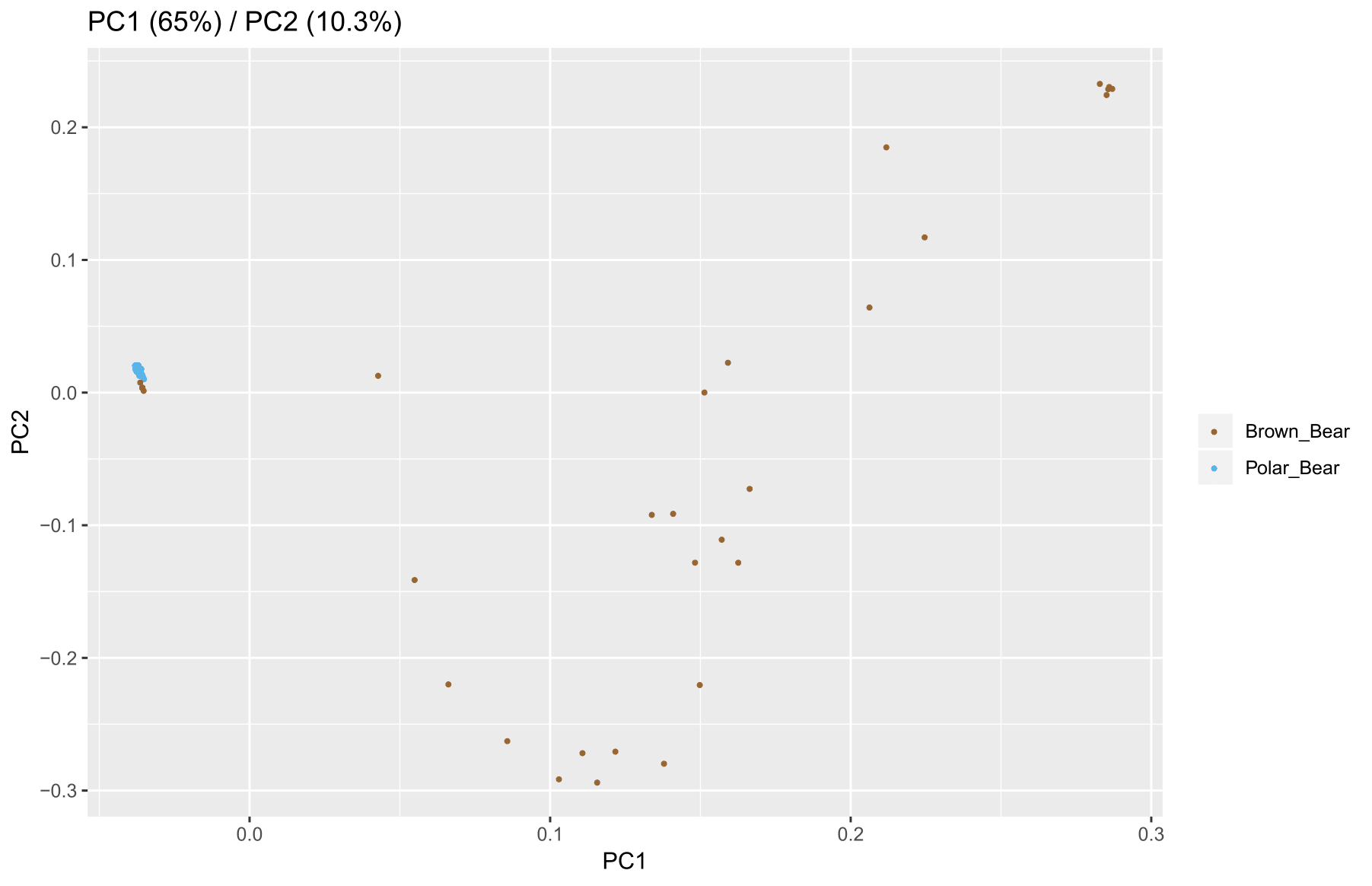
**

**Supplementary figure 6:** Principal component analysis of EHD3 and the 50kb flanking regions using all individuals included in this study.

**
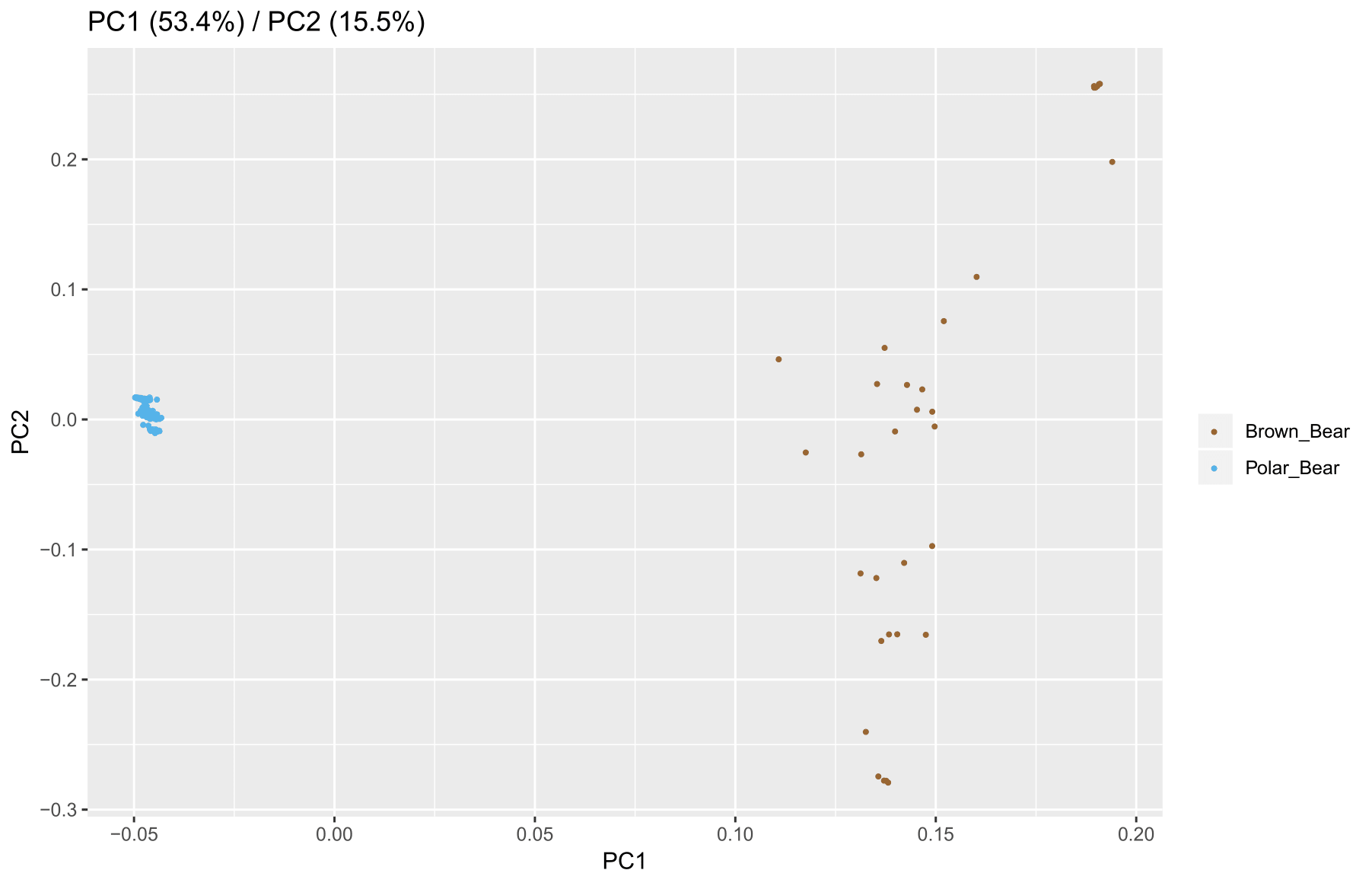
**

**Supplementary figure 7:** Principal component analysis of FCGBP and the 50kb flanking regions using all individuals included in this study.

**
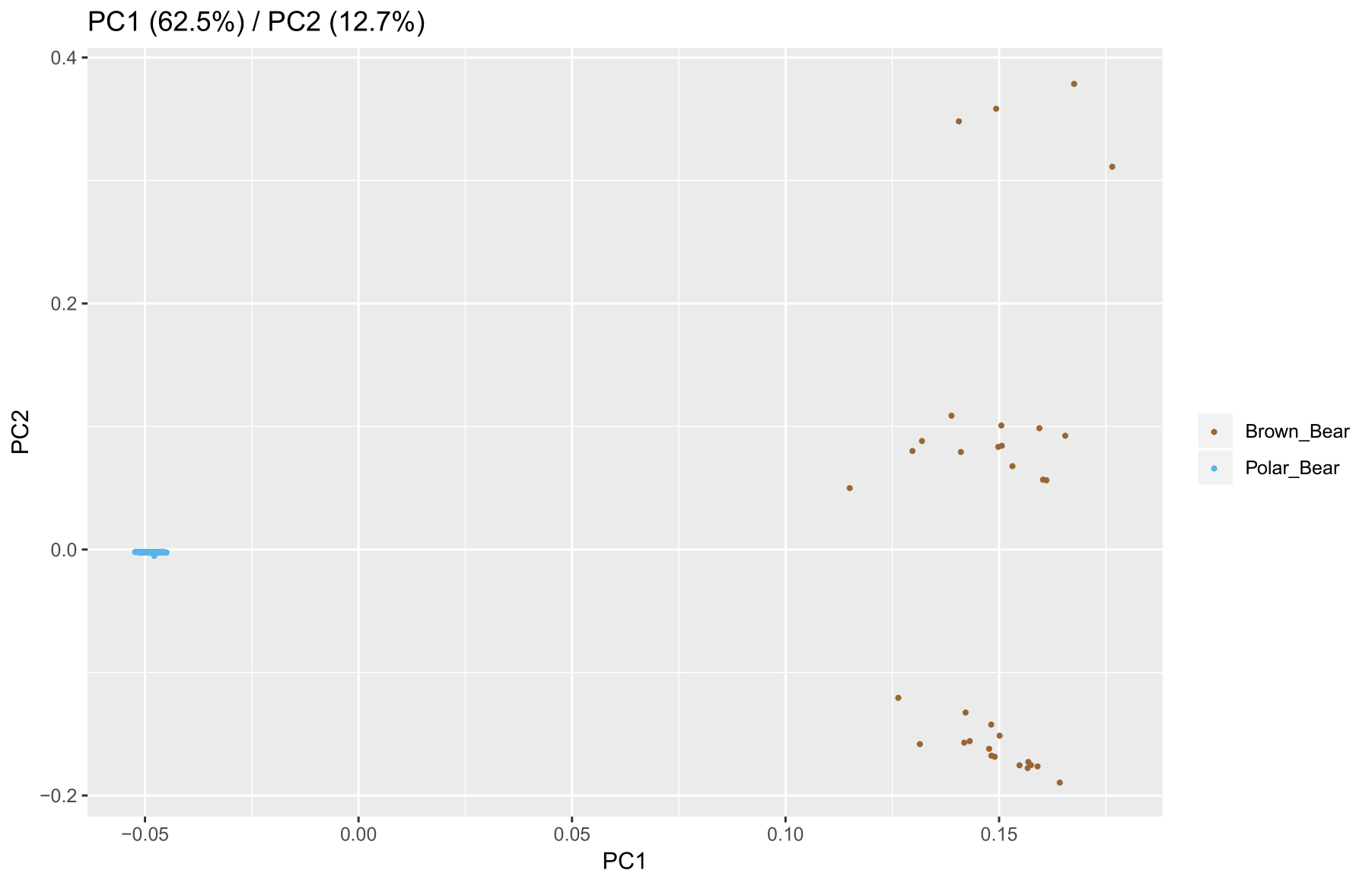
**

**Supplementary figure 8:** Principal component analysis of LAMC3 and the 50kb flanking regions using all individuals included in this study.

**
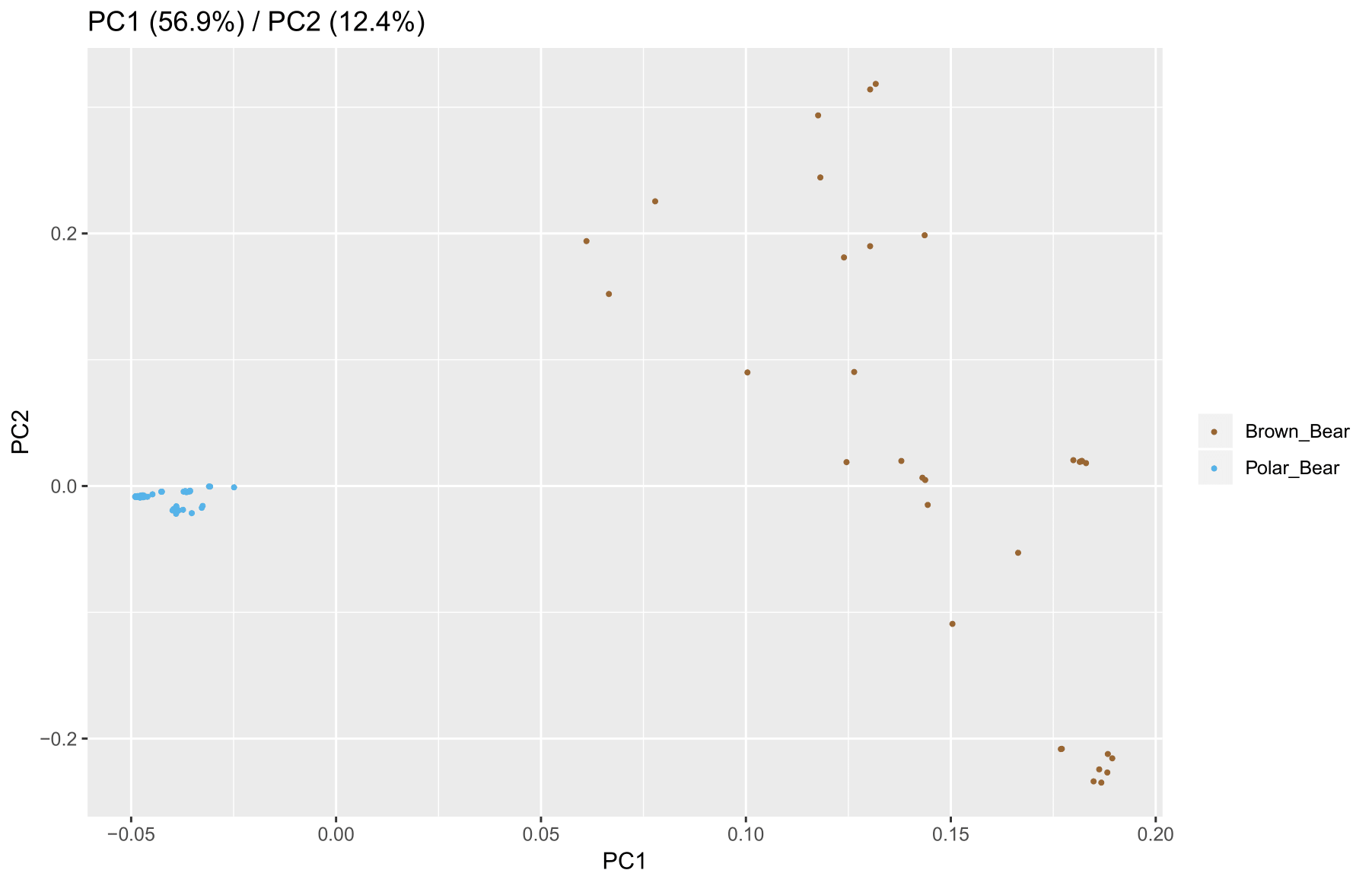
**

**Supplementary figure 9:** Principal component analysis of LYST and the 50kb flanking regions using all individuals included in this study.

**
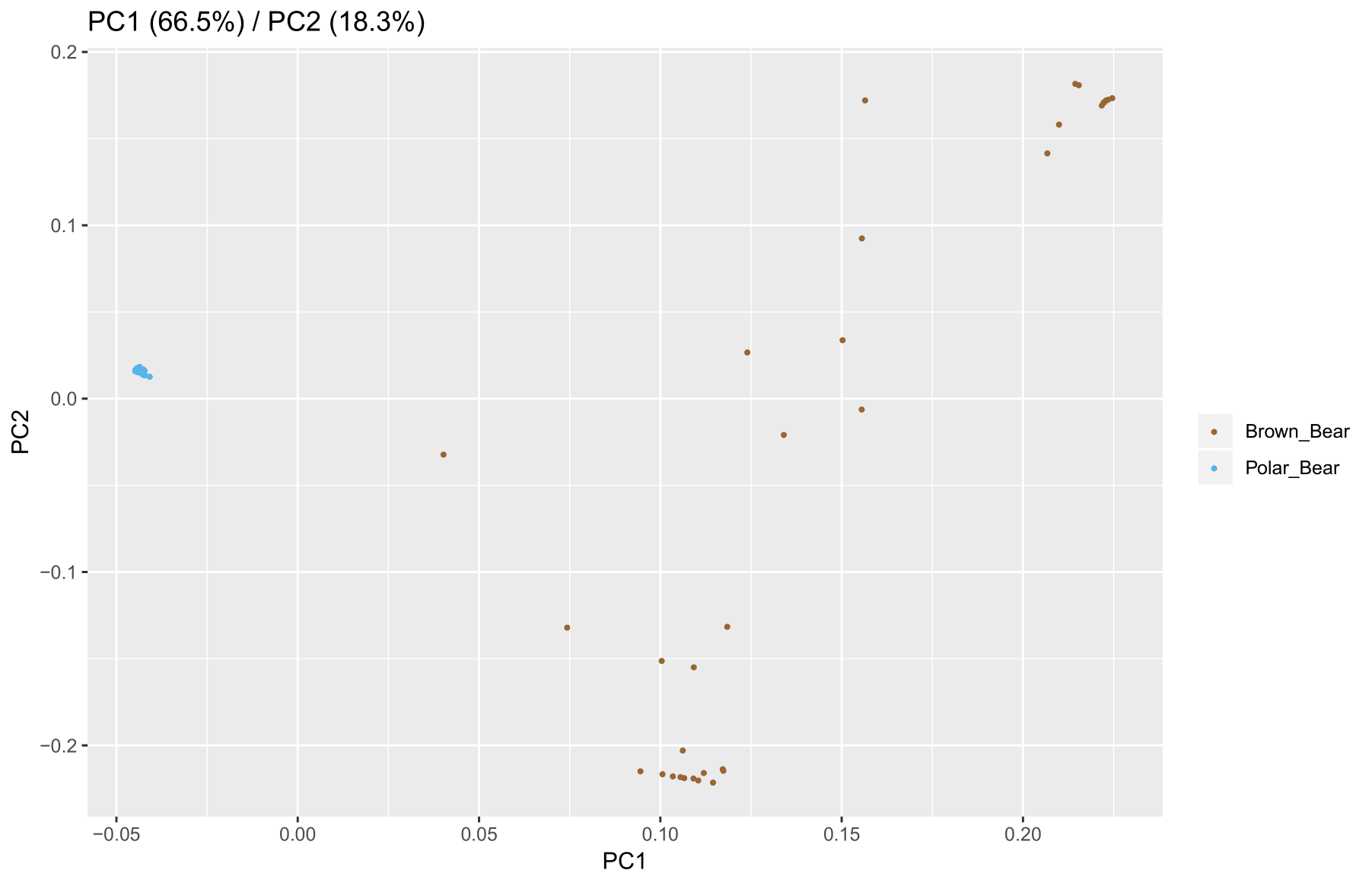
**

**Supplementary figure 10:** Principal component analysis of POLR1A and the 50kb flanking regions using all individuals included in this study.

**
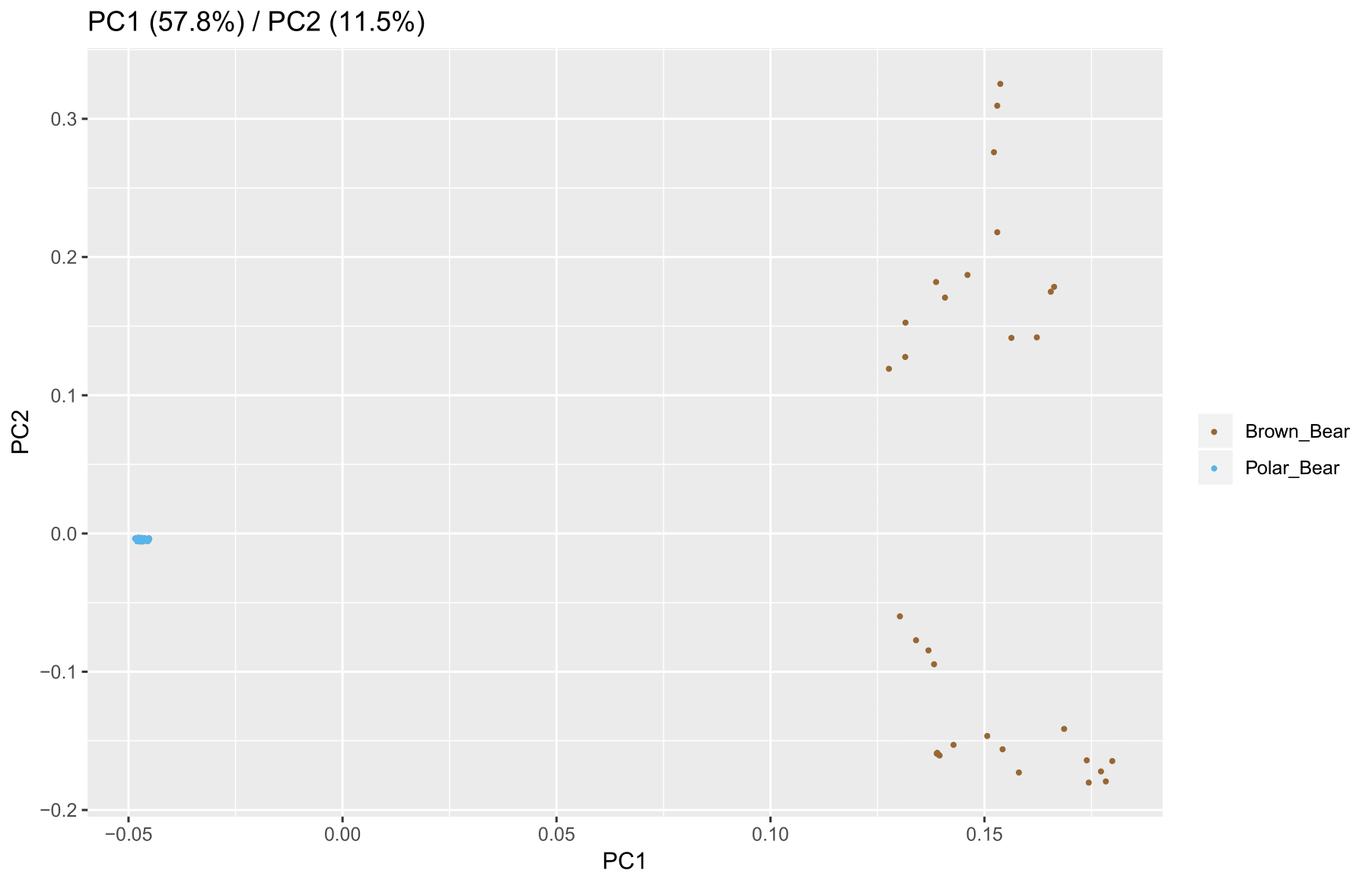
**

**Supplementary figure 11:** Principal component analysis of TTN and the 50kb flanking regions using all individuals included in this study.

**
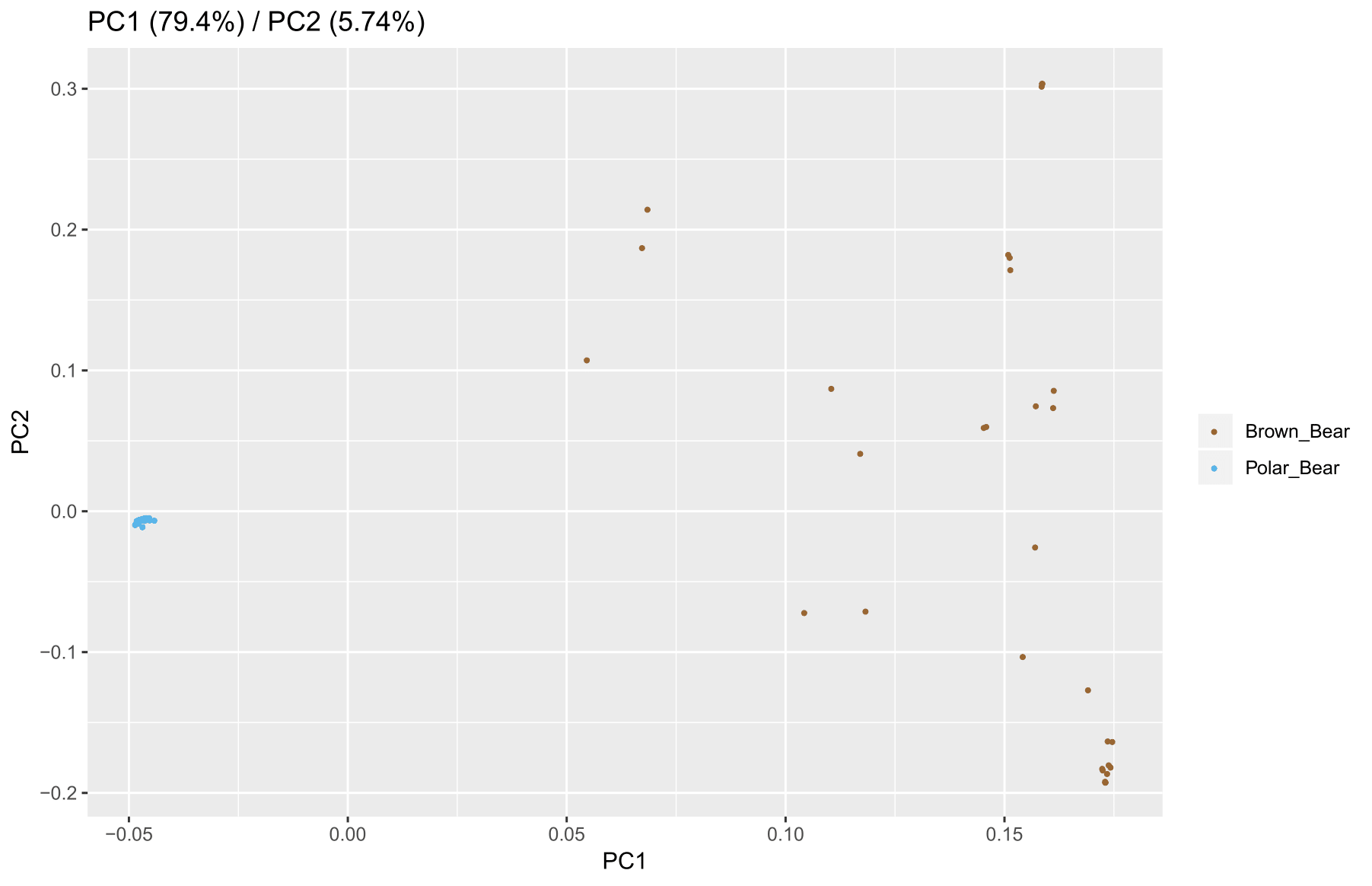
**

**Supplementary figure 12:** Principal component analysis of XIRP1 and the 50kb flanking regions using all individuals included in this study.
